# Supplementary material for: The kinetic profiles of copeptin and mid regional proadrenomedullin (MR-proADM) in pediatric lower respiratory tract infections
Source: PLoS One. 2022 Mar 10;17(3):e0264305. doi: 10.1371/journal.pone.0264305 (PMC8912143; doi:10.1371/journal.pone.0264305)
Supplement: S1 Appendix — (DOCX) [file pone.0264305.s001.docx]

**S1 Appendix**

**Methods**

**Nonlinear mixed effect modelling approach**

The non-linear mixed effects modeling approach is a one stage analysis that simultaneously estimates fixed effect parameters, inter-individual variability, and random residual error.

In addition to provide the average behavior of the group (the mean plasma concentration-time course), it provides an estimate of variability on parameters, identify its sources and quantify the unexplained part of variability. Thus, such models take into account both explained and unexplained variability at inter- and intra-individual levels.

Non-linear mixed effect models are characterized in terms of:

1. Fixed effects: this is the population average of the model parameters θ. These parameters are susceptible to various factors, such as clinical and demographic characteristics, inflammatory biomarkers, disease state, treatment, etc. These last factors are the fixed effect covariates, z_i_.
2. Random effects: this is the part of the variability that is not explained by the above fixed effect and allows quantification of:
   - The *inter-individual* variability which is the variability between two different individuals. It is expressed by ω^2^, which is the variance of the fixed effect parameter θ. For an individual I,

$\theta_{i}=\theta\cdot\exp\left( \eta_{i} \right)$, with $\eta_{i}\sim N\left( 0,\omega^{2} \right)$

- The *intra-individual* variability (also called the residual unexplained variability), which is the variability within the same individual over time i.e. between two given moments. It is expressed by σ^2^. For an observation j, the corresponding prediction ŷ for an individual is $y_{i,j}$ = ŷ + $\varepsilon_{i,j}$, with $\varepsilon_{i,j}\sim N(0,\sigma^{2})$

Thus, the general mixed effects model is written:

$\boldsymbol{y}_{\boldsymbol{i,j}}$ **=** $\boldsymbol{f}\left( \boldsymbol{x}_{\boldsymbol{ij}}\boldsymbol{,}\boldsymbol{\phi}_{\mathbf{i}} \right)\boldsymbol{+}\boldsymbol{\varepsilon}_{\boldsymbol{i,j}}$

And the parameter model:

$\boldsymbol{\phi}_{\mathbf{i}}$ **= g**$\left( \boldsymbol{z}_{\boldsymbol{i}}\boldsymbol{,}\boldsymbol{\theta} \right)\boldsymbol{+}\boldsymbol{\eta}_{\boldsymbol{i}}$

Where $y_{ij}$ is the j^th^ observation in an individual i, $x_{ij}$ is a known quantity (time), $\phi_{i}$ is the parameter vector for an individual i, and $\varepsilon_{ij}$ represents the residual error; g is a structural model which is function of fixed effects covariates z_i_, and fixed effects parameters θ; finally, $f$ represents the structural model.

Thereby, three parameters have to be estimated:

- the fixed effect vector: $\theta_{i}$ according to individual covariate(s) z_i_ identified to influence model parameters θ
- the random effect parameter quantifying the residual unknown variability: $\sigma^{2}$
- the random effect parameter quantifying the inter-individual variability: $\Omega$.

*Base model:*

Different structural models were tested according to the distribution observed in the description phase. In the equation y is the dependent variable (copeptin and MR-proADM), β_0_ is the intercept (baseline or starting concentration at day 1 of inclusion, pmol/L for copeptin and nmol/L for MR-proADM), β_1_ the slope (in day^-1^) for concentration change over time (t, in day), $\beta_{lim}$ the concentration plateau achieved at the end of the study:

Linear

$y= \beta_{0}+\beta_{1}t$ (1)

Log-linear

$\log\left( y \right)= \beta_{0}+\beta_{1}t \equiv y=\beta_{0}\cdot{exp}^{(\beta_{1}\cdot t)}$ (2)

Log-linear with a lower limit β_lim_ (pmol/L for copeptin and nmol/L for MR-proADM)

$y=\beta_{lim}+(\beta_{0}-\beta_{lim})\cdot{exp}^{(\beta_{1}\cdot t)}$ (3)

The fixed parameters to be estimated are $\beta_{0}$*,* $\beta_{1}, \beta_{lim}$

Between-subject variability (random effect) is included initially on each parameter to be estimated ($\beta_{0}$, $\beta_{1}$, $\beta_{lim}$) and assumed to be log-normally distributed. Additive distribution could be considered according to initial results. (e.g. to account for increasing levels in some patients). Proportional, additive and combined (proportional and additive) error model were investigated to account for residual unexplained variability (random effect).

Correlation between parameters was tested.

- - - *Boeckmann A, Sheiner L, Beal S. Introductory guide. NONMEM Users Guide. University of California at San Fransisco: NONMEM Project Group. 1994.*
    - *Mould DR, Upton RN. Basic concepts in population modeling, simulation, and model-based drug development. CPT Pharmacometrics Syst Pharmacol. 2012;1:e6.*

**Parameter Estimation and Model Selection**

Estimation was performed using the Laplace first-order conditional estimation with interaction (FOCE-I/LAPLACE). The likelihood ratio test, based on the difference in objective function value (ΔOF) was used to compare two nested models during forward and backward procedure. The Akaike Information Criterion (AIC) was used for non-nested models. For both criteria, a lower value corresponds to a better fit. For nested model, since -2 log likelihood, approximate χ2 distribution, it was considered statistically significant if it exceeded 3.8 (p<0.05) and 6.6 (p<0.01) points, for one additional parameter during model-building and backward deletion procedures, respectively. Model assessment was also based on goodness-of-fit plots along with precision of the model parameters estimates, and the reduction of inter- and intra- individual variability.

Sensitivity analysis was performed if patients presented with absolute values for conditional weighted residuals (CWRES) greater than 6 to test for potential bias in parameter estimation and in covariate exploration.

*Bayon et al. Establishing Best Practices and Guidance in Population Modeling: An Experience With an Internal Population Pharmacokinetic Analysis Guidance CPT: Pharmacometrics & Systems Pharmacology (2013) 2, e51; doi:10.1038/psp.2013.26*

**Influence of patient characteristics (covariates search)**

Association with clinical parameters, laboratory variables, patient management, microbiology and chest radiography, called covariates (COV), were investigated on model parameters (P) on which variability was identified. Selected covariates are described in S1 Table and the stepwise covariate model building approach is explained in S1 Appendix. . Covariates were included in the model following a sequential forward selection (selected if p-value<0.05) and backward elimination (kept if p-value<0.01). Continuous covariates were tested for potential relationship using linear (4) and power (5) parameterization:

$P= \theta_{1}\cdot\left( \frac{COV}{{COV}_{median}} \right)^{\theta_{2}}$ (4)

$P=\theta_{1}.\left( 1+\theta_{2}.(COV-{COV}_{median} \right)$ (5)

Categorical covariates were tested as followed (6):

$P=\theta_{1}.\left( 1+\theta_{2}.COV \right)$ (6)

where $\theta_{1}$is the typical value of the parameter P (baseline $\beta_{0}$, or slope $\beta_{1}$ or lower limit $\beta_{\lim})$ for the typical patient i.e. when the covariate is equal to the reference value and $\theta_{2}$ is the estimated parameter describing the magnitude of the covariate-parameter relationship, P is the individual estimated model parameter and COV is individual covariate value

**Model evaluation**

The final model stability was assessed by the bootstrap method using the PsN-Toolkit. (version 3.5.3, Uppsala, Sweden). The median and the 95% confidence interval (95%CI) estimated from 2000 re-sampled data sets were compared to the original model estimations. In addition, visual predictive checks (VPC) were performed with PsN-Toolkit and Xpose4 (version 4.3.5, Uppsala, Sweden) to assess the predictive performance of the model. VPC were obtain from 200 simulations of the data with parameter estimates from base and final model. Simulated 10th, median, and 90th percentiles,

and their 95%CIs were compared with observed values.

**Results**

**NLME: Base model**

At study initiation, between subject variability was high in both copeptin and MR-proADM concentrations, >100% and 74% respectively. On the parameter$slope \beta_{1}$, describing the decrease in biomarker levels, between subject variability was of 46% and 43% for copeptin and MR-proADM respectively. The variability in copeptin levels achieved at the end of the study (parameter $\beta_{lim}$) was of 67%; Between subject variability on parameter $\beta_{lim}$ could not be identified for MR-proADM. Additive distribution provided a better fit on slope parameter $\beta_{1}$ for MR-proADM while a log-normal distribution was used for coeptin.

**Influence of patient characteristics on copeptin kinetics**

Copeptin covariate model building showed signs of overparameterization. Convergence was in most case not obtained. Thus, none of the studied variable could be included in the model to explain variability in copeptin concentrations.

**Table 1. Copeptin model parameter estimates with final model associated bootstraps.**

|  | Base and final model | | | Bootstraps analysis  (n = 500 samples) | | |
| --- | --- | --- | --- | --- | --- | --- |
| Parameter | Estimate | RSE (%) | %CV | Estimate | 2.5th percentile | 97.5th percentile |
| Copeptin day 1 $\beta_{0}$ (pmol/L) | 7.85 | 7 | 118.5 | 7.83 | 6.84 | 9.15 |
| Copeptin decrease $\beta_{1}$ (/day) | -0.995 | 35 | 46 | -0.998 | -2.105 | -0.470 |
| Limit $\beta_{lim}$ (pmol/L) | 5.49 | 7 | 67.3 | 5.425 | 4.65 | 6.06 |
| Proportional residual error (%) | 31.8 | 5 |  | 31.7 | 28.7 | 34.9 |

RSE: Relative Standard Error; %CV: coefficient variation.

At the population level, copeptin concentration at baseline were estimated at 7.85 pmol/L, decrease over studies with a slope estimated at -0.995/day to reach a concentration of 5.49 pmol/L at the end of the study.

**Influence of patient characteristics on MR-proADM**

During forward covariate model building, antibiotic administration, general complication, microbiology results and presence of fever had a significant influence on MR-proADM on day 1 (p<0.001). Admission to ICU and microbiology results were associated with the the steepness of MR-proADM decrease. During the backward process, association with fever became not significant and was thus excluded.

Based on visual inspection oral administration and no administration of antibiotic were grouped*.* Administration of intravenous (IV) antibiotic remained associated to different baseline levels. On slope parameters, microbiology classified as no blood culture performed and classified as other microbiological findings showed similar slope estimates and were pooled together. Thus, a first model was obtained where baseline was higher for patients receiving IV antibiotics, was lower in patients with negative NPA or no growth in BC or in patients with no BC performed and higher in patients with pneumococcus/streptococcus positive BC as compared to patients classified as other for microbiology, and baseline was higher in patients with complication. Slope was lower in patients admitted to ICU as compared to patients not admitted to ICU and higher in patients with negative NPA or no growth in BC and Pneumococcus/Streptococcus positive BC as compared to the other microbiology classification. Bootstraps performed on this model showed that the influence of complications on baseline was not significant and neither was the influence of Pneumococcus/Streptococcus positive BC and “BC not performed” t (2.5^th^ – 97.5^th^ percentile includes 0). Thus, these covariates were excluded from the model. The reduced model was estimated and bootstraps were performed again. Estimates for negative microbiology results were close to 0 and bootstraps showed that it was no more significant. Model was again reduced to exclude the influence of any microbiology results on baseline.

The final model is presented in Table 2. It retains the association antibiotic IV administration with baseline, and the association of ICU admission and negative NPA or no growth in BC and Pneumococcus/Streptococcus positive BC with slope.

**Table 2. MR-proADM: Model parameter estimates with final model and associated bootstraps**

|  | Base model | | | Final model | | | Bootstraps analysis  (n = 500 samples) | | |
| --- | --- | --- | --- | --- | --- | --- | --- | --- | --- |
| Parameter | Estimate | RSE (%) | %CV | Estimate | RSE (%) | %CV | Estimate | 2.5th percentile | 97.5th percentile |
| MR-proADM (nmol/L) on day 1 | 0.49 | 3.5 | 77.4 | 0.43 | 3 | 70.8 | 0.43 | 0.40 | 0.45 |
| AB-IV |  |  |  | 0.61 | 20 |  | 0.61 | 0.39 | 0.90 |
| Decrease (/day) | -0.42 | 23 | 43.0 | -0.30 | 19 | 32.3 | -0.30 | -0.49 | -0.21 |
| ICU admission |  |  |  | -1.07 | 21 |  | -1.03 | -1.50 | -0.43 |
| No growth in BC or Pneumococcus/Streptococcus positive BC |  |  |  | 0.85 | 28 |  | 0.85 | 0.45 | 1.44 |
| Limit (nmol/L) | 0.25 | 9 | - | 0.23 | 8 | - | 0.23 | 0.19 | 0.27 |
| Proportional residual error (%) | 29.3 | 6 |  | 28.8 | 6 |  | 28.6 | 25.3 | 31.9 |

AB-IV: intravenous antibiotics; RSE: Relative Standard Error; %CV: coefficient variation of inter-individual variability; ICU: intensive care unit; BC: blood culture.

Baseline = $0.43\times(1+0.61\times ABIV)$

Slope = -0.30 $\times(1-1.07\times ICUadmission)\times(1+0.85 Microbio No growth or\frac{\mathrm{Pneumococcus}}{\mathrm{Streptococcus}})$

Limit = 0.23

At the population level, for the typical patient, MR-proADM concentration at baseline were estimated at 0.43 nmol/L, decrease over studies with a slope estimated at -0.30/day to reach a concentration of 0.23 nmol/L at the end of the study. The typical patient is defined as not receiving antibiotic IV, not being admitted at ICU and where BC was not performed.

Patient that received IV antibiotics presented an increase baseline by 61%. Patient for which was BC performed (with either positive or negative BC results) lead to decrease slope by 0.85/day and patient that were admitted to ICU lead to increase slope by 1.07/day
