# Supplementary material for: The kinetic profiles of copeptin and mid regional proadrenomedullin (MR-proADM) in pediatric lower respiratory tract infections
Source: PLoS One. 2022 Mar 10;17(3):e0264305. doi: 10.1371/journal.pone.0264305 (PMC8912143; doi:10.1371/journal.pone.0264305)
Supplement: S1 Fig — Age-stratification and change in MR-proADM (nmol/L), and copeptin (pmol/L) concentrations over the study days 1, 3, and 5. (DOCX) [file pone.0264305.s002.docx]

**S1 Fig. Age Group and Biomarkers**


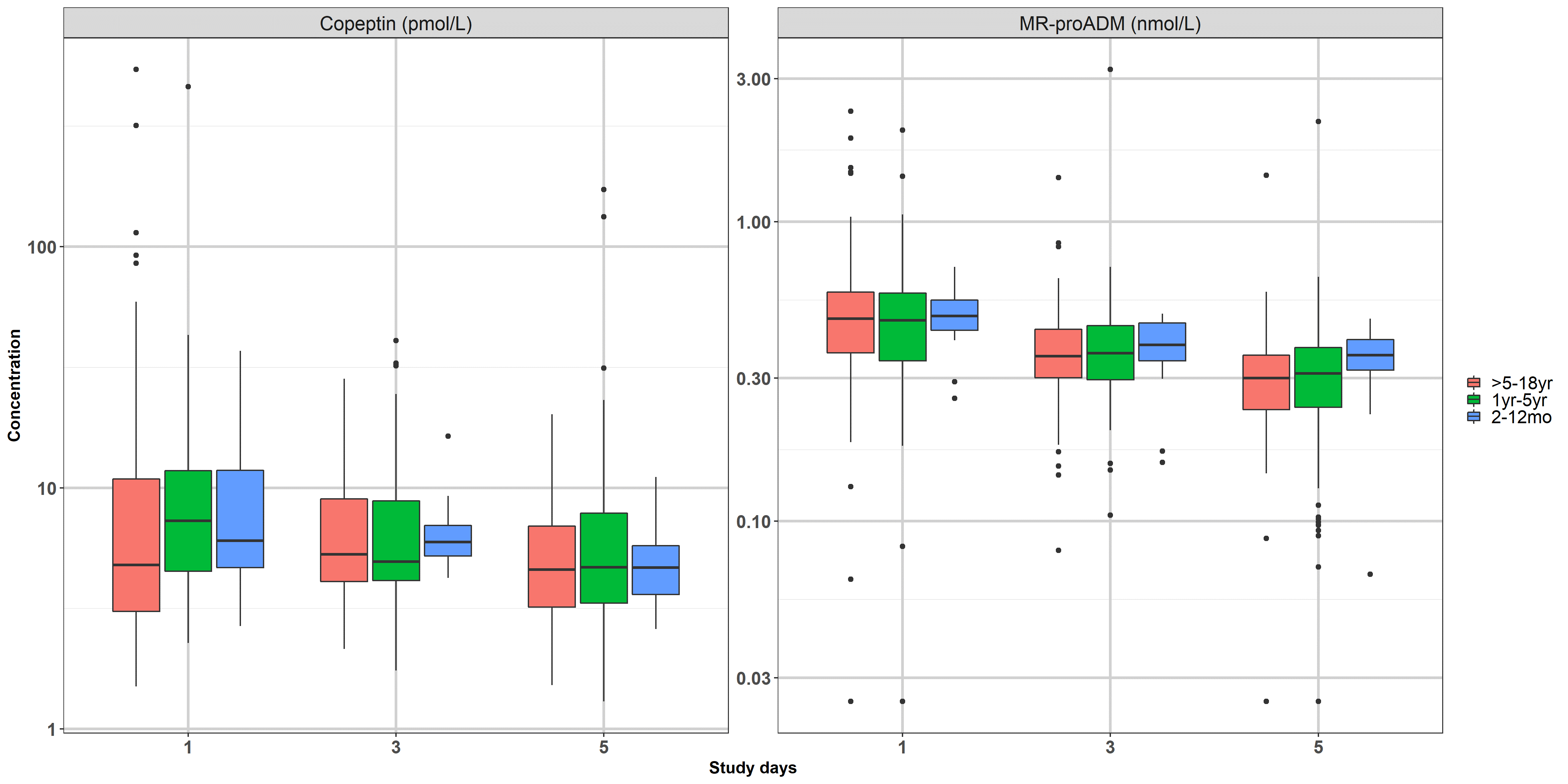


Age-stratification and change in copeptin (pmol/L) and MR-proADM (nmol/L) concentrations over the study days 1, 3, and 5.
