# Supplementary material for: The kinetic profiles of copeptin and mid regional proadrenomedullin (MR-proADM) in pediatric lower respiratory tract infections
Source: PLoS One. 2022 Mar 10;17(3):e0264305. doi: 10.1371/journal.pone.0264305 (PMC8912143; doi:10.1371/journal.pone.0264305)
Supplement: S1 Table — Classification as normal or elevated rate were set according Fleming S, Thompson M, Stevens R et al. Normal ranges of heart rate and respiratory rate in children from birth to 18 years of age: a systematic review of observational studies. The Lancet. 2011; 377(9770):1011±8 and WHO. (DOCX) [file pone.0264305.s003.docx]

**S1 Table. Cut-off values to define normal or elevated breath and heart rates according to patient’s age.**

| **Age group** | **Higher limit set for breath rate** | **Higher limit set for heart rate** |
| --- | --- | --- |
| < 2 months | 60 /min | 160 b/min |
| 2-12: months | 50 /min | 140 b/min |
| 1 to 5 years | 40 /min | 120 b/min |
| > 5 years |  | 90 b/min |

Classification as normal or elevated rate were set according Fleming S, Thompson M, Stevens R et al. Normal ranges of heart rate and respiratory rate in children from birth to 18 years of age: a systematic review of observational studies. The Lancet. 2011; 377(9770):1011±8 and WHO.
