# Supplementary material for: The kinetic profiles of copeptin and mid regional proadrenomedullin (MR-proADM) in pediatric lower respiratory tract infections
Source: PLoS One. 2022 Mar 10;17(3):e0264305. doi: 10.1371/journal.pone.0264305 (PMC8912143; doi:10.1371/journal.pone.0264305)
Supplement: S2 Table — Age-stratification and change in MR-proADM (nmol/L), and copeptin (pmol/L) concentrations over the study days 1, 3, and 5. SD: Standard deviation. (DOCX) [file pone.0264305.s004.docx]

**S2 Table. Age Group and Biomarkers.**

| **Biomarker** | **Age Group** | **Day** | **Median** | **Percentile 25** | **Percentile 75** | **Min** | **Max** | **Mean** | **SD** |
| --- | --- | --- | --- | --- | --- | --- | --- | --- | --- |
| MR-proADM (nmol/L) | 5-18yr | 1 | 0.47 | 0.36 | 0.58 | 0.02 | 2.33 | 0.57 | 0.39 |
| MR-proADM (nmol/L) | 5-18yr | 3 | 0.36 | 0.3 | 0.44 | 0.08 | 1.4 | 0.39 | 0.19 |
| MR-proADM (nmol/L) | 5-18yr | 5 | 0.3 | 0.24 | 0.36 | 0.02 | 1.43 | 0.32 | 0.17 |
| MR-proADM (nmol/L) | 1yr-5yr | 1 | 0.47 | 0.34 | 0.58 | 0.02 | 2.02 | 0.5 | 0.27 |
| MR-proADM (nmol/L) | 1yr-5yr | 3 | 0.36 | 0.3 | 0.45 | 0.1 | 3.22 | 0.4 | 0.33 |
| MR-proADM (nmol/L) | 1yr-5yr | 5 | 0.31 | 0.24 | 0.38 | 0.02 | 2.16 | 0.32 | 0.24 |
| MR-proADM (nmol/L) | 2-12mo | 1 | 0.48 | 0.43 | 0.55 | 0.26 | 0.71 | 0.49 | 0.12 |
| MR-proADM (nmol/L) | 2-12mo | 3 | 0.39 | 0.34 | 0.46 | 0.16 | 0.49 | 0.37 | 0.1 |
| MR-proADM (nmol/L) | 2-12mo | 5 | 0.36 | 0.32 | 0.4 | 0.07 | 0.47 | 0.34 | 0.1 |
| Copeptin (pmol/L) | 5-18yr | 1 | 4.8 | 3.07 | 10.88 | 1.5 | 543.89 | 24 | 74.79 |
| Copeptin (pmol/L) | 5-18yr | 3 | 5.29 | 4.09 | 9 | 2.15 | 28.35 | 7.18 | 5.36 |
| Copeptin (pmol/L) | 5-18yr | 5 | 4.58 | 3.21 | 6.95 | 1.52 | 20.19 | 5.64 | 3.63 |
| Copeptin (pmol/L) | 1yr-5yr | 1 | 7.3 | 4.51 | 11.77 | 2.27 | 461.93 | 15.14 | 49.22 |
| Copeptin (pmol/L) | 1yr-5yr | 3 | 4.94 | 4.13 | 8.82 | 1.75 | 40.8 | 7.51 | 6.53 |
| Copeptin (pmol/L) | 1yr-5yr | 5 | 4.68 | 3.33 | 7.85 | 1.3 | 172.53 | 9.99 | 22.88 |
| Copeptin (pmol/L) | 2-12mo | 1 | 6.04 | 4.68 | 11.81 | 2.67 | 36.97 | 10.53 | 9.76 |
| Copeptin (pmol/L) | 2-12mo | 3 | 5.98 | 5.23 | 7 | 4.23 | 16.38 | 6.77 | 2.98 |
| Copeptin (pmol/L) | 2-12mo | 5 | 4.67 | 3.61 | 5.75 | 2.6 | 11.1 | 5.26 | 2.43 |

Age-stratification and change in MR-proADM (nmol/L), and copeptin (pmol/L) concentrations over the study days 1, 3, and 5. SD: Standard deviation.
