# Supplementary material for: The kinetic profiles of copeptin and mid regional proadrenomedullin (MR-proADM) in pediatric lower respiratory tract infections
Source: PLoS One. 2022 Mar 10;17(3):e0264305. doi: 10.1371/journal.pone.0264305 (PMC8912143; doi:10.1371/journal.pone.0264305)
Supplement: S3 Table — ICU: intensive care unit; ARDS: acute respiratory distress syndrome. (DOCX) [file pone.0264305.s005.docx]

**S3 Table. Type and day of complication experienced by five patients during the 5 study days.**

| **Patient** | **Day** | **Type of complication** | **ICU admission** |
| --- | --- | --- | --- |
| 1 | 3 | Sepsis | No |
| 2 | 3 | Sepsis+pleural effusion+empyema | Yes |
|  | 5 | ARDS+sepsis+empyema |  |
| 3 | 3 | ARDS+sepsis+pleural effusion | Yes |
|  | 5 | ARDS+sepsis+pleural effusion |  |
| 4 | 3 | Sepsis | No |
|  | 5 | Sepsis |  |
| 5 | 5 | Pleural effusion | Yes |

ICU: intensive care unit; ARDS: acute respiratory distress syndrome.
