# Supplementary material for: The kinetic profiles of copeptin and mid regional proadrenomedullin (MR-proADM) in pediatric lower respiratory tract infections
Source: PLoS One. 2022 Mar 10;17(3):e0264305. doi: 10.1371/journal.pone.0264305 (PMC8912143; doi:10.1371/journal.pone.0264305)
Supplement: S4 Table — MR-proADM: pro-adrenomedullin; IQR: inter-quartile range. (DOCX) [file pone.0264305.s006.docx]

| **S4 Table. Copeptin, and MR-proADM concentrations and relative change between study days.** | | | | | | |
| --- | --- | --- | --- | --- | --- | --- |
|  | **Concentrations,**  **median (IQR)** | | | **Relative change,**  **median (IQR), %** | | |
|  | **Day 1** | **Day 3** | **Day 5** | **Day 1 to  Day 3** | **Day 3 to  Day 5** | **Day 1 to  Day 5** |
| **MR-proADM (**nmol/L) | 0.47  (0.36 - 0.58) | 0.36  (0.3 – 0.45) | 0.31  (0.24 – 0.37) | -23.4  (-16.7;-22.4) | -13.9  (-20;-17.8) | -34  (-33.3;-33.2) |
| **Copeptin (**pmol/L) | 6.26  (3.87 - 11.66) | 5.23  (4.22 – 8.82) | 4.65  (3.3 – 7.27) | -16.5  (+9; -24.4) | -11.1  (-21.8 ; -17.6) | -25.7  (-14.7;-37.7) |

MR-proADM: pro-adrenomedullin; IQR: inter-quartile range.
