# Supplementary material for: The kinetic profiles of copeptin and mid regional proadrenomedullin (MR-proADM) in pediatric lower respiratory tract infections
Source: PLoS One. 2022 Mar 10;17(3):e0264305. doi: 10.1371/journal.pone.0264305 (PMC8912143; doi:10.1371/journal.pone.0264305)
Supplement: Attachment — Submitted filename: Reviewers comments for PLOS ONE JOURNAL.docx [file pone.0264305.s007.docx]

Reviewer’s comments on manuscript submitted to PLOS ONE

Manuscript number- PONE-D-20-08646

Title- Copeptin and mid regional proadrenomedullin (MR-proADM) in pediatric lower respiratory infections

General comments

The authors of this manuscript reported the possible roles of two novel biomarkers (copeptin and MR-proADM) in predicting disease severity in pediatric lower respiratory infections (LRTIs). It was a post-hoc analysis of a randomized controlled trial evaluating procalcitonin (PCT) as a biomarker guiding antibiotic treatment in children and adolescents with LRTIs in the ProPAED trial. Biomarker research aimed at improving disease diagnosis and prognostication remains a relevant issue in scientific literature. Although the authors gave an in-depth report of their study which made a case for MR-proADM alone or in combination with PCT, cytokines or C-reactive protein (CRP) in predicting disease severity in LRTIs, there are few specific concerns that I request them to address to enhance the manuscript.

Specific comments

1. Abstract and Title: The full title of your manuscript does not convey the main objective of the study. Curiously, the title is the same as your short title. Based on the aim of your study, I suggest a modification of your full title to read- ‘The kinetic profiles of copeptin and mid-regional proadrenomedullin in pediatric lower respiratory tract infections.’ In the background of your abstract,	you mentioned that both novel biomarkers may be predictive of complications of pediatric LRTIs. Which complications are you referring to? Again, the phrase-‘The change over time of these biomarkers during LRTIs’ may better be rendered thus- ‘The kinetics of these biomarkers during LRTIs.’ The second part of the aim read- ‘…and to investigate the relationship between copeptin, MR-proADM and demographic, clinical and laboratory characteristics of pediatric LRTI.’ I suggest you rephrase this for clarity.
2. Introduction: Study aims stated in lines 79-84 appear slightly different from the aims under Abstract. Can you please harmonize them?
3. Methods: Every study methodology should have clarity and easy reproducibility as key features. Why did you adopt a post-hoc analysis of an RCT in your study? Post-hoc analysis conducted and interpreted without sufficient consideration of the ‘multiple testing problem’ is criticized by some scholars who believe the statistical associations are often spurious. In fact, you to admitted this methodological flaw of using a retrospective approach in one of your listed study limitations (lines 428-430)
4. Discussion: There are many redundant statements in your discussion which made it difficult to follow and understand. For instance, there are repetition of results in several paragraphs. I suggest a discussion with this frame work for clarity and conciseness: the research problem or gap and the significance of addressing or filling it (paragraph 1), critical analysis of your major findings (paragraph 2), additional findings and how they fit into the existing literature (paragraph 3), study limitations (paragraph 4), future directions (paragraph 5) and overall conclusion and major impact of the study (paragraph 6). Your study limitations may appear illogical to some readers. In lines 423-425, you tend to suggest that severity of the presentation of LRTIs is determined by the location of medical practice. However, it is obvious there are underlying conditions such as immunosuppression that could worsen disease presentation and serve as confounders. In any case, your study exclusion criteria lent credence to this fact (lines 94-95).
