# Supplementary material for: The kinetic profiles of copeptin and mid regional proadrenomedullin (MR-proADM) in pediatric lower respiratory tract infections
Source: PLoS One. 2022 Mar 10;17(3):e0264305. doi: 10.1371/journal.pone.0264305 (PMC8912143; doi:10.1371/journal.pone.0264305)
Supplement: Attachment — Submitted filename: Response to Reviewers.docx [file pone.0264305.s008.docx]

Pediatric Infectious Disease and Vaccines	Yu Ru Kou, PhD

University Children’s Hospital Basel	Academic Editor

Spitalstrasse 33	*PLOS ONE*

Postfach	1265 Battery Street

4031 Basel, Switzerland	Suite 200

**Prof. Dr. Jan Bonhoeffer, Vice-Chair**	San Francisco

T +41 61 704 12 12	CA 94111

jan.bonhoeffer@ukbb.ch	USA

**Dr. Philipp Baumann, Research Fellow**

T +41 44 266 70 76

M +41 76 73 46 977

F +41 44 266 71 68

philipp.baumann@kispi.uzh.ch

Basel, January 30, 2022

**Revised Manuscript** **PONE-D-20-08646R1: The kinetic profiles of copeptin and mid regional proadrenomedullin (MR-proADM) in pediatric lower respiratory tract infections**

Dear Yu Ru Kou,

thank you very much for considering the publication of our manuscript. Here we answer point-by-point to the aspects raised by reviewer #1. Please see the corresponding corrections in the uploaded manuscript which we think is now suitable for publication. All line numbers in the revised manuscript correspond to the track-change version.

*Reviewer #1: Please abbreviate the 95% confidence intervals as 95%CI (not CI95%) in the abstract and everywhere else in the text.*

Authors` response: “CI95%” has been replaced by “95%CI” in Manuscript in lines 42, 43, and 46, and also in S1 Appendix.

*Please replace all mentions of 'multivariate' analysis with 'multivariable' - all analyses here focused on one outcome at a time, not multiple outcomes in the same model - they are therefore multivariable NOT multivariate.*

Authors` response: “Multivariate” has been replaced by “multivariable” in Manuscript in lines 197/198 (Table 2 caption) and in Table 2 itself.

*Please add 95% confidence intervals to the estimates of effect listed in Table 2 and wherever else they are cited in the text.*

Authors` response: 95%CIs have been inserted into Table 2 as requested.

We are grateful to the reviewer and editors at PLOS ONE for the work they have done on our manuscript. Please feel free to contact us anytime if further questions should arise.

Kind regards,

Philipp Baumann
